# Human Arthropod-Borne Viral Infections

**DOI:** 10.1155/2013/608510

**Published:** 2013-12-31

**Authors:** Aldo Manzin, Byron E. Martina, Ernest A. Gould, Patrizia Bagnarelli, Vittorio Sambri

**Affiliations:** ^1^Department of Biomedical Sciences, Clinical Microbiology and Virology Unit, University of Cagliari, Policlinico Monserrato, S.S. 554, Bivio per Sestu, 09042 Monserrato, Cagliari, Italy; ^2^Department of Viroscience, Erasmus Medical Center, Room Ee 1714a's Gravendijkwal 230, 3015 CA Rotterdam, The Netherlands; ^3^UMR190, Emergence des Pathologies Virales Aix-Marseille Universite, Institut de Recherche pour le Développement, Ecole des Hautes Etudes en Santé Publique, Unité des Virus Emergents, Faculté de Médecine de Marseille, 27 Boulevard Jean Moulin, 13005 Marseille Cedex 05, France; ^4^Virology Unit, Department of Biomedical Sciences and Public Health, Università Politecnica delle Marche, Via Tronto 10/a, 60020 Torrette di Ancona, Ancona, Italy; ^5^Unit of Clinical Microbiology, The Greater Romagna Area-Hub Laboratory, 60 Piazza della Liberazione, 47522 Pievesestina (FC), Italy

Arthropod-borne viruses, that is, arboviruses, belong to different virus families and genera and are maintained through transmission between vertebrate hosts by virus-infected blood feeding arthropods (mosquitoes, sandflies, biting midges, and ticks). Several factors have contributed to the emergence and reemergence of arthropod-to-human infections in the last decade, also in nonendemic areas: climate changes which are believed to have contributed to increased dispersion of the virus vectors; the international trading system that transports an enormous range of goods, animals, and human beings all over the world, inadvertently increasing the possibility for vectors and viruses to spread; and the global increase of human population density, particularly in areas densely populated by arthropods with transmission capability for several different pathogenic viruses. As a consequence, the incidence of arboviral infections and diseases is increasing worldwide and therefore placing a heavier burden on public health agencies and requiring the need to ensure timely recognition and treatment of virus infections.

This special issue is aimed at the presentation of recent results obtained by prominent colleagues in the field of human infections caused by vector-borne viruses. Faced with a limited number of manuscripts presented, the high quality of these papers will contribute significantly to our knowledge of these important issues.

Pathogens, such as dengue virus, Japanese encephalitis virus, and alphaviruses are addressed by the authors. G. Añez and M. Rios described the epidemiology of what is likely to be the most widespread mosquito transmitted *Flavivirus* infection, that is, dengue fever, in the United States of America where this infection is currently generating huge concerns. In particular, the authors emphasize how, despite the current economic difficulties and restrictions, strict mosquito control policies and activities must be implemented and maintained in localities that have the potential to become the route of entry for viruses and the focus of epidemics.

A second paper by C.-F. Liu and coworkers reported on the complementary role of clinical practice-based laboratory data in facilitating suspicion and diagnosis of dengue in clinical settings, thus ameliorating the overall diagnostic capabilities for this infection. Although the authors state that further studies are needed to confirm the validity of their approach, we believe that the study is important to define which procedures will have the most significant impact on clinical practice in tropical countries, where medical resources are limited. R. Rodriguez-Roche and E. A. Gould contributed a comprehensive review about the complex epidemiology and pathogenesis of the dengue viruses. They underlined the need for more effort to understand and accelerate the development of suitable vaccines and/or antiviral therapies for the control of this “scourge” of the tropical and subtropical world. Another paper by M. L. Muñoz and coworkers is dedicated to the investigation of the interplay between dengue virus proteins and the receptor polypeptides located on the surface of mosquito cells, since this interaction is the fundamental basis for viral entry into susceptible cells, followed by development and transmission in the vectors. The paper by I.-K. Lee and coworkers provides an insight into the immunopathogenesis of dengue virus in patients with type 2 diabetes mellitus and reports on the ability of mononuclear cells from these patients to be infected by dengue virus. The aim of the paper is to fill a gap in our knowlwdge that concerns epidemiological data and immunological findings of frequent dengue haemorrhagic fever and Th2 cytokines in patients with diabetes mellitus. The authors demonstrated that *in vitro* infection of mononuclear cells from diabetes mellitus patients induces higher levels of both IL-4 and the anti-inflammatory Th2-cytokine IL-10, in addition to increased levels of GM-CSF, and they speculated that these findings might result from a counterbalance to the comparatively highly activated proinflammatory cytokines/chemokines in the diabetic hosts.

Two papers describe different aspects of Japanese encephalitis virus (JEV) related diseases. The paper by G. Kakoti and coworkers reports on the clinical profile and the outcome of Japanese encephalitis virus infection in children up to 12 years old. This is a prospective study based on serological assays and it shows some points of interest from a clinical and epidemiological point of view as it investigates the presence of clinical or technical factors likely to be significantly correlated with increased mortality by JEV in children with acute encephalitis syndrome in North East India. B. Bandyopadhyay and collaborators reported the epidemiology of JEV in West Bengal (India) showing that 22.76% and 5% of the acute encephalitis syndrome cases were positive for JE IgM in 2011 and 2012, respectively, and that JE is mainly prevalent in children and adolescents below 20 years of age with no gender predilection. This is a significant observation from an epidemiological point of view, which shows how active surveillance of JE cases is still warranted. It also emphasizes the need for vigilance to identify the introduction of new genotypes in the endemic areas or to detect evidence of dispersion into newer geographical districts.

This series of papers is completed by a paper by I. Assunção-Miranda that describes the molecular mechanisms involved in the pathogenesis of alphavirus-induced arthritis, the paper by A. Burgueño and coworkers that investigated the seroprevalence of Saint Louis encephalitis related antibodies among horses in Uruguay, where no sharp epidemiological data about this virus were available so far, and the article by G. Sautto and collaborators that speculated about the possible use of candidate monoclonal antibodies to be possibly used in a future passive immunotherapy for arthropod-borne infections. This approach seems of particular importance, given the current absence of specific antiviral drugs as well as effective vaccines for the most diffused arbovirus infections.

